# Detection of Pre-Impact Falls from Heights Using an Inertial Measurement Unit Sensor

**DOI:** 10.3390/s20185388

**Published:** 2020-09-20

**Authors:** Youngho Kim, Haneul Jung, Bummo Koo, Jongman Kim, Taehee Kim, Yejin Nam

**Affiliations:** Department of Biomedical Engineering, Yonsei University, Wonju 26493, Korea; hanul1219@ybrl.yonsei.ac.kr (H.J.); bmk726@ybrl.yonsei.ac.kr (B.K.); jmkim0127@ybrl.yonsei.ac.kr (J.K.); kth940923@ybrl.yonsei.ac.kr (T.K.); namyj1007@ybrl.yonsei.ac.kr (Y.N.)

**Keywords:** falls from heights, wearable airbag, complementary filter, inertial measurement unit, detection algorithm

## Abstract

Many safety accidents can occur in industrial sites. Among them, falls from heights (FFHs) are the most frequent accidents and have the highest fatality rate. Therefore, some existing studies have developed personal wearable airbags to mitigate the damage caused by FFHs. To utilize these airbags effectively, it is essential to detect FFHs before collision with the floor. In this study, an inertial measurement unit (IMU) sensor attached to the seventh thoracic vertebrae (T7) was used to develop an FFH detection algorithm. The vertical angle and vertical velocity were calculated using the inertial data obtained from the IMU sensor. Forty young and healthy males were recruited to perform non-FFH and FFH motions. In addition, experiments using a human mannequin and dynamics simulations were performed to obtain FFH data at heights above 2 m. The developed algorithm achieved 100% FFH detection accuracy and provided sufficient lead time such that the airbags could be inflated completely before collision with the floor.

## 1. Introduction

Industrial and construction sites have hazardous working environments where many labor-intensive activities are performed, and several accidents can occur. Accidents in industrial sites include falls, occupational accidents, falls from heights (FFHs), fractures, and crush injury. Among these, falls and FFHs occur most frequently. According to the Korea Industrial Accident Analysis Data for 2018 [1], falls and FFHs account for 18.65% and 15.21% of the safety accidents that occur in industrial sites, respectively. In particular, the number of fatalities from FFHs was 376, accounting for one-third of the total fatalities in industrial sites. In 2014, Christina et al. [2] reported that FFHs constitute the second most common cause of work-related injuries and fatalities, resulting in 300,000 injuries and 818 traumatic deaths in the US. According to data from the Bureau of Labor Statistics [3], the number of fatal injuries due to FFHs increased by 26% from 2011 to 2016. The Health and Safety Executive Annual Statistics [4] reported that 40 of the 147 industrial site deaths in Great Britain in 2018/2019 were due to FFHs, accounting for the highest rate of deaths in industrial sites. The International Labour Organization [5] also mentioned that the fatality rate observed for FFHs is the highest among industrial accidents, and this issue should be handled carefully.

Thus, to prevent safety accidents such as FFHs in industrial sites, various training programs have been implemented to provide safety training to workers and site managers. Kaskutas et al. [6] conducted 8 h safety training sessions that included safety communication and fall protection for foremen and apprentices and evaluated the effectiveness of the training sessions through worksite observational audits and surveys. Consequently, the number of unsafe motions was reduced, and the number of times FFH prevention regulations were followed increased. Marin et al. [7] implemented a 5 h safety training program that included FFH prevention, silica exposure, leadership, communication, and safety planning for 118 workers. Six months later, the program was evaluated by Leaders in Safe Construction through surveys. The survey results showed that 58% of educated people were able to communicate effectively, 52% were more responsible for maintaining safety, and 62% said the program was effective in preventing safety accidents. However, despite efforts such as conducting safety training programs, the number of FFHs has still increased [3,8,9].

The recommended physical measures to prevent FFHs include installing guardrails, covers, and safety nets and wearing personal fall arrest systems (PFASs) [10]. Chi et al. [11] analyzed various FFHs according to gender, age, working periods, and company sizes and reported that guardrails and safety nets can help prevent FFHs and minimize the damage caused by FFHs. Bobick et al. [12] evaluated the safety of two commercially available guardrails based on the criteria provided by the Occupational Safety and Health Administration and determined that the guardrails meet the safety requirements for FFH prevention. However, guardrails, covers, and safety nets are mainly installed on the outskirts of the workplace; consequently, these measures cannot prevent FFHs caused by roof or scaffold collapses within the workplace. PFASs can be used anywhere in the workplace by hanging their hooks; however, suspension trauma [13] can occur as a result of the hanging rope, and PFASs cannot prevent FFHs caused by the collapse of the workplace itself. In addition, PFASs can interrupt the movements of workers, and they do not function effectively at heights below 15 ft [14].

To address the limitations of existing prevention methods, some studies have proposed the use of personal wearable airbags to mitigate the impact caused by FFHs on the human body [15,16,17,18]. The airbags were usually worn as clothes but were inflated to protect the human body like car airbag when dangerous situations were detected. These wearable airbags offer superior impact attenuation effects compared with commercially available protective pads [19]. Detecting FFHs before collision with the floor is essential for inflating these airbags to utilize them effectively. FFHs can be detected by either using visual devices, such as cameras, or using wearable sensors. Han et al. [20] used a Microsoft Kinect sensor and an iPi Soft Motion Capture solution and captured 90.91% of the unsafe movements performed when climbing a ladder. Shrestha et al. [21] obtained video data using high-resolution closed-circuit television (CCTV) cameras installed in the workplace. They tracked the locations of workers and determined whether the workers wore a hard hat. The system was designed to send a warning alarm when it was determined that a worker was not wearing a hard hat. Fang et al. [22] attempted to collect image data from the workplace using the mask-region-based convolution neural network and identified whether the workers moved on structural supports. The system obtained a 90% precision rate and a 75% recall rate. However, systems that use visual devices are not effective because workers might be obscured by various structures in the workplace. In addition, existing FFH detection systems that use visual devices are difficult to be applied in wearable airbags because these systems detect dangerous situations with a high probability of FFHs occurring, instead of detecting the actual FFHs.

Only a few studies have been reported on FFH detection with wearable sensors. Dogan et al. [23] performed free-fall experiments of an accelerometer. They classified FFH using root mean square (RMS) values of acceleration and calculated the fall height with an error of 10.8% using falling time. Yang et al. [24] attached an inertial measurement unit (IMU) to the waist of two participants; classified FFH and non-FFH motions such as walking, squatting, standing, losing balance, and near-fall activities on a steel frame using a support vector machine (SVM); and reported an FFH detection accuracy of 93.8%. Dzeng et al. [25] used RMS values of acceleration from the smartphone to detect FFH. Four experimental participants performed some motions during tiling jobs, such as squatting, standing, and falling. They obtained 100% sensitivity and 96.1% specificity for FFH detection. However, the number of participants and experimental motions was not sufficient to objectively evaluate FFH detection systems in previous studies using wearable sensors. Moreover, the systems used the data at collision period and thus could not be for pre-impact FFH detection.

Many studies have been conducted for the detection of pre-impact falls of the elderly. In addition, there are many public datasets available online for fall detection systems (UMAFall [26], SisFall [27], and UP-Fall [28]). Pre-impact fall detection systems have been developed based on machine learning (ML) and threshold-based approaches. Tong et al. [29] presented a hidden Markov model (HMM)-based approach to detect and predict fall events by using the upper trunk’s triaxial accelerations obtained from eight healthy young student volunteers. They acquired a lead time of 200–400 ms and 100% accuracy. Zhen et al. [30] calculated the RMS of acceleration and angle using inertial data from the waist and developed a pre-impact fall detection algorithm using an SVM. The algorithm obtained 99% sensitivity, 96.5 specificity, and an average lead time of 268 ms. In general, ML-based fall detection algorithms showed good performance, but it took considerable time to extract features and to train classifiers. Therefore, it is difficult to use an ML-based pre-impact fall detection algorithm in real time.

On the other hand, for the threshold-based algorithms, Wu et al. [31] calculated the vertical velocity using inertial data obtained from the front waist and developed a pre-impact fall detection algorithm. Their algorithm achieved 100% sensitivity, three false alarms (the number of non-falls detected as falls), and a lead time of over 70 ms. Nyan et al. [32] also developed a threshold-based pre-impact fall detection algorithm using angles calculated from the torso and thigh (with two IMUs), as well as the correlation coefficients of the torso and thigh angles. The algorithm achieved 100% specificity, 95.2% specificity, and a lead time of 700 ms. Sabatini et al. [33] estimated the vertical velocity and height change using an IMU with a barometric altimeter to detect fall and achieved 80% sensitivity, 100% specificity, and an average lead time of 157 ms. Jung et al. [34] performed experiments on 30 healthy young males and obtained inertial data from the back waist. A fall detection algorithm was developed using acceleration RMS, angular velocity RMS, and vertical angle (VA). They achieved 98.33% accuracy and a lead time of 280.25 ± 10.29 ms with their own data and 92.4% accuracy with SisFall public datasets. 

Public datasets for the detection of fall of the elderly exist, and some previous studies detected pre-impact falls of the elderly with good performance. However, it is necessary to develop an algorithm for the detection of FFHs in worksites, which are different from falls of the elderly. FFH in worksites occurring from great heights is more fatal than fall of the elderly. In industrial sites, harsh motions such as shoveling and pickaxing and jump motion from relatively low heights are performed. Therefore, algorithms for the detection of fall of the elderly would not work in the detection of FFH in worksites.

In this study, a pre-impact FFH detection algorithm that can be applied in wearable airbags was developed to overcome the limitations of existing systems. Forty healthy young males participated in two types of experiments in which they performed various non-FFH motions and one FFH motion. In addition, in order to obtain inertial data on FFHs from great heights that cannot be used on humans owing to safety concerns, we conducted experiments using a human mannequin and dynamics simulations. The vertical angle (VA) and vertical velocity (VV) were calculated using the inertial data from the experiments and simulations, and were used as parameters in the FFH detection algorithm. The VA was calculated using a complementary filter with a proportional integral (PI) controller. The VV was calculated by integrating the vertical acceleration obtained using the Euler angle transformation of the three-axis acceleration data.

## 2. Materials and Methods 

### 2.1. Experimental Participants

Two types of experiments involving humans were conducted to obtain inertial data. Forty healthy young male students from Yonsei University participated in the experiments. Among the 40 participants, 20 (age: 24.8 ± 1.4 years; height: 174.1 ± 4.9 cm; weight: 73.7 ± 10.1 kg) participated in Experiment 1, and 20 (age: 25.8 ± 2.0 years; height: 173.5 ± 6.1 cm; weight: 76.6 ± 13.0 kg) in Experiment 2. The Yonsei University Research Ethics Committee (1041849-201911-BM-166-01, 1041849-202004-BM-042-02) approved the experiments, and they were conducted after obtaining written consent from all the participants.

### 2.2. Equipment

An MPU-9250 [35] (InvenSense, San Jose, CA, USA) sensor capable of measuring three-axis acceleration (±16 g), angular velocity (±2000°/s), and magnetism (±4800 μT) was used as the IMU sensor. An nRF52832 chip (Nordic Semiconductor, Trondheim, Norway) was used to receive the inertial data from MPU-9250 through I2C communication and transmit the same to a PC via radio frequency (2.4 GHz) communication. LabVIEW (National Instruments, Austin, TX, USA) was used to save the data on a PC as a .csv file. A webcam (SPC-A1200MB, Samsung, Seoul, Korea) and a 3D motion capture system (Vicon Motion Systems Ltd., Oxford, UK), which were synchronized with the IMU sensor sampled at 100 Hz, were used to record the video and marker data.

### 2.3. Experimental Procedures

**Figure 1:**
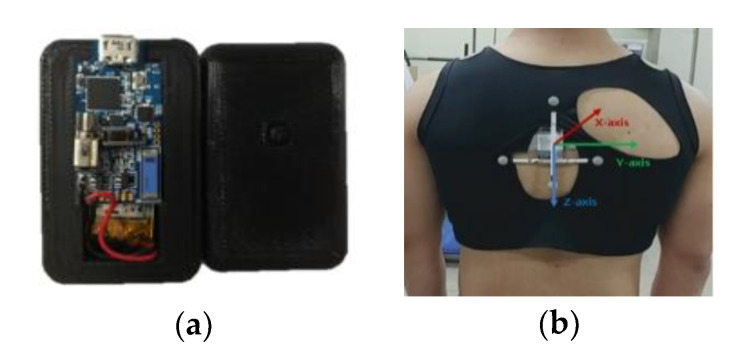
(**a**) IMU and (**b**) sensor position.

The IMU sensor was positioned on the seventh thoracic vertebrae (T7) (Figure 1). The axes of the IMU were set such that the anteroposterior was along the x-axis, mediolateral along the y-axis, and superoinferior along the z-axis. 

**Figure 2:**
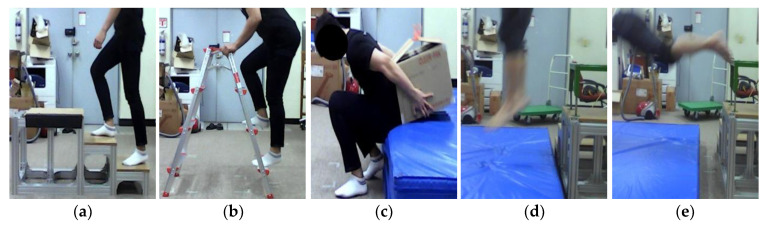
Examples of experimental motions: (**a**) climbing up and down stairs, (**b**) climbing up and down a ladder, (**c**) lifting (back), (**d**) 0.7 m jump, and (**e**) 0.7 m forward FFH.

In Experiment 1, the participants performed nine types of non-FFH motions and one FFH motion, three times each (Table 1). In Experiment 2, the participants performed six types of non-FFH motions, in addition to the motions performed in Experiment 1 (Table 2). Figure 2 shows some of the motions performed in the experiments. 

Experiments 1 and 2 were performed to obtain data to develop an FFH detection algorithm and evaluate the algorithm, respectively. We divided the experiments into two in order to evaluate the developed algorithm with the new data that were not used in the algorithm development process and with the new motions’ data that were not included in Experiment 1.

### 2.4. Mannequin Experiments

**Figure 3:**
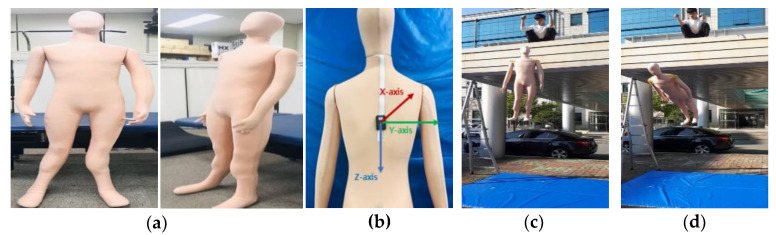
(**a**) Mannequin used in the experiments, (**b**) sensor position, (**c**) vertical FFH, and (**d**) forward FFH.

In experiments involving humans, it was not possible to perform FFH motions at great heights for safety reasons. Thus, to obtain inertial data on FFHs from great heights, we used a human mannequin (Madamade, Gyeonggi-do, Korea) (height: 180 cm; weight: 10 kg) made of soft and durable knit fabric to withstand the impact. Motions involving 0.7 m jump and 0.7 m forward FFH were performed to compare the data obtained using the mannequin with those obtained from humans. Since the mannequin cannot jump, the 0.7 m jump was replaced by a vertical free-fall motion at a height of 0.7 m. Then, vertical and forward FFH motions were performed at 2, 2.5, and 3 m. All the motions were performed five times. The IMU sensor was positioned as in the experiments involving humans. Figure 3 shows the mannequin used in the experiments, the position of the sensor, and examples of the mannequin experiments. 

### 2.5. Dynamics Simulations

FFH motions were performed at great heights in the mannequin experiments; however, it was difficult to perform the FFH motions observed in actual industrial sites due to limited space and lack of equipment. Therefore, dynamics simulations were performed to obtain data on the actual FFH motions in industrial sites. Pam-Crash (ESI Group, Paris, France) and a human articulated rigid body (HARB) model (height: 170 cm; weight: 80 kg) were used. Motions involving 0.7 m jump and 0.7 m forward FFH were performed to compare the data obtained from dynamics simulations with those obtained from experiments involving humans. Since the HARB model could not jump, the 0.7 m jump was replaced by a vertical free-fall motion at a height of 0.7 m. 

**Figure 4:**
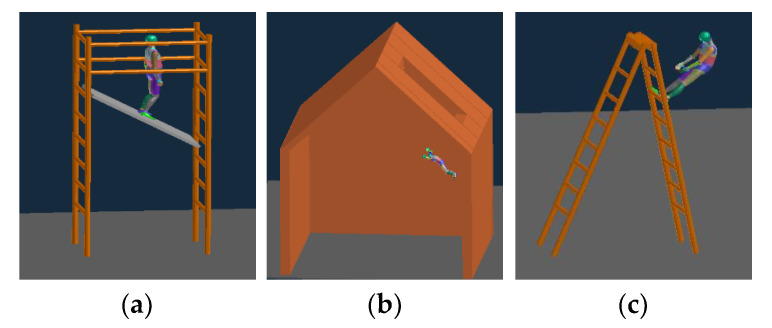
FFH motions in the dynamics simulations: (**a**) scaffold FFHs, (**b**) roof FFHs, and (**c**) ladder FFHs.

Zlatar et al. [36] analyzed 114 types of FFHs according to height, situation, and degree of injury. They reported that the most frequent FFHs occurred at heights between 3 and 6 m, and many FFHs occurred when working on scaffolds, roofs, and ladders. Dynamics simulations were performed based on these findings to obtain data on FFH motions when working on scaffolds, roofs, and ladders at heights of 3, 4.5, and 6 m. The inertial data were obtained at the same positions as those in the experiments involving humans. Figure 4 shows the motions in the dynamics simulations.

### 2.6. Data Analysis

MATLAB R2019a (MathWorks Inc., Natick, MA, USA) was used to analyze the experimental data and to develop and evaluate the FFH detection algorithm. A 5 Hz low-pass filter was used to filter the inertial data obtained from the IMU sensor to remove high-frequency noise.

**Figure 5:**
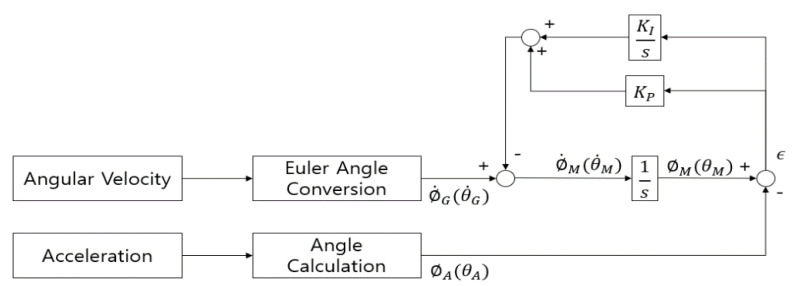
Vertical angle (VA) calculation using a complementary filter with a PI controller.

The VA was calculated using a complementary filter with a PI controller [34] to minimize integration errors and the effect of external impacts. Figure 5 shows the flowchart of a complementary filter with the PI controller. The difference between the VA values calculated using the complementary filter with the PI controller and those obtained from the 3D motion capture system was 5.2 ± 3.2°.

The VV was obtained by integrating the vertical acceleration. The vertical acceleration was calculated by the Euler angle transformation of the three-axis acceleration data [37]. Only the vertical acceleration component, $a_{z,g}$, was used, with g added to make the vertical acceleration equal to 0 g in the static state. The equations are as follows:

(1) $$\left[\begin{array}{c} a_{x,g}\\ a_{y,g}\\ a_{z,g} \end{array}\right]=\left[\begin{array}{ccc} cos\theta & sin\theta sin\emptyset & sin\theta cos\emptyset \\ 0 & cos\emptyset & -sin\emptyset \\ -sin\theta & cos\theta sin\emptyset & cos\theta cos\emptyset \end{array}\right]\left[\begin{array}{c} a_{x,l}\\ a_{y,l}\\ a_{z,l} \end{array}\right],$$

(2) $$a_{v}=a_{z,g}+1g,$$

In Equation (1), $a_{x,g},\;a_{y,g},\;\mathrm{and}\;a_{z,g}$ represent the accelerations along the x-, y-, and z-axes in the global coordinates, respectively; $a_{x,l},\;a_{y,l},\mathrm{and}\;a_{z,l}$ represent the accelerations along the x-, y-, and z-axes in the local coordinates, respectively. ∅(roll) and θ(pitch) represent the VAs in the frontal and sagittal planes, respectively. $a_{v}$ represents the vertical acceleration.

The VV can be obtained by integrating the vertical acceleration, but an integral accumulation error occurs when simple integration is performed. Lee et al. [38] used a conditional damping-based integration technique to remove this error. In their study, the vertical acceleration and jerk reference value were selected through trial and error; if a value greater than the reference value was entered, then integration was executed. In this study, we used a modified version of the above technique. Integration was executed using Simpson’s rule when the vertical acceleration was 0.24 g or more. If the vertical acceleration was less than 0.24 g, then the VV was initialized to 0 m/s with an attenuation ratio of 0.9. We selected a vertical acceleration of 0.24 g experimentally through trial and error. The estimated VV was lower than the actual VV because integration was not performed from the beginning owing to the conditional integration technique. The VV values calculated from the IMU sensor data during free-fall tests at heights of 1, 1.5, and 2 m were compared with those obtained from the 3D motion capture system, and a correction factor of 1.2 was used. The final VV calculation was performed as follows:

(3) $$v_{v,t}=\{\begin{array}{l} if\;a_{v}\;>\;0.24g,\\ v_{v,t-2}+1.2g\left(a_{v,t-2}+4a_{v,t-1}+a_{v,t}\right)\Delta t/3\\ otherwise,\\ v_{v,t-1}*0.9 \end{array},$$

where $v_{v}$ represents the VV.

The VV calculated from the inertial data and that obtained from the 3D motion capture system were compared for the experiments involving humans to validate the VV calculated using the above equation. To confirm whether there were significant differences between the two sets of values, we performed a one-sample *t*-test (*p* < 0.05) using IBM SPSS Statistics 25 (IBM, New York, NY, USA). The results indicated that there were no significant differences between the two sets of values.

### 2.7. Algorithm Development

To set thresholds for the VA and VV to be used in the FFH detection algorithm, we used data collected from 10 randomly selected participants in Experiment 1. In a general receiver operating characteristic (ROC) curve, the x- and y-axes represent specificity and sensitivity, respectively. In this study, a modified ROC curve was used in which the x- and y-axes represented VV threshold and accuracy, respectively. Sensitivity, specificity, and accuracy were calculated as follows:

(4) $$\mathrm{Sensitivity}\;\left(\%\right)=\frac{TP}{TP\;+\;FN}\times 100,$$

(5) $$\mathrm{Specificity}\;\left(\%\right)=\frac{TN}{TN\;+\;FP}\times 100,\;$$

(6) $$\mathrm{Accuracy}\;\left(\%\right)=\frac{TP\;+TN}{TP\;+TN\;+\;FP\;+\;FN}\times 100,$$

where TP(true positive) is the number of FFHs detected as FFHs, FN(false negative) is the number of FFHs detected as non-FFHs, TN(true negative) is the number of non-FFHs detected as non-FFHs, and FP(false positive) is the number of non-FFHs detected as FFHs.

**Figure 6:**
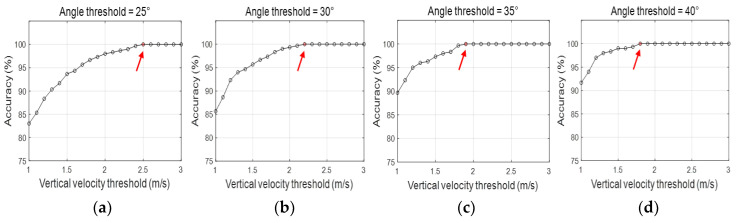
Accuracy determined using VA and VV thresholds.

The threshold VA values were set to 25°, 30°, 35°, and 40°, and the threshold VV values were set from 1 to 3 m/s, with intervals of 0.1 m/s for each VA threshold. Then, accuracy was calculated according to the threshold combinations of the VA and VV (Figure 6). Four threshold combinations of the VA and VV (25°, 2.5 m/s; 30°, 2.2 m/s; 35°, 1.9 m/s; and 40°, 1.8 m/s) were obtained with 100% accuracy.

Data collected from the remaining 10 participants were used to select the best threshold combination among the four combinations. Table 3 presents the sensitivity, specificity, and accuracy levels obtained using the threshold combinations.

The lead time was determined to select one of the threshold combinations (35°, 1.9 m/s; 40°, 1.8 m/s). The lead time is the interval between the collision time and the FFH detection time. It was calculated using the following equation:

Lead time = Collision time − FFH detection time.(7)

Table 4 presents the lead times of FFHs according to the threshold combinations (35°, 1.9 m/s; 40°, 1.8 m/s). The threshold combination with a longer lead time (40°, 1.8 m/s) was selected as the final threshold combination for the FFH detection algorithm.

**Figure 7:**
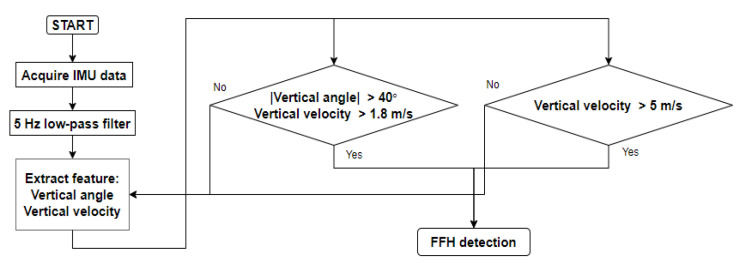
Flowchart of the threshold-based algorithm for detecting FFHs.

An algorithm using only the VV was necessary as the algorithm containing the VA could not detect FFHs in which the VA did not change. In the algorithm using only the VV, the threshold was set to 5 m/s; this was greater than the maximum VV of non-FFHs, namely, 4.14 m/s. Figure 7 shows the flowchart of the final algorithm developed for detecting pre-impact FFHs.

## 3. Results

The algorithm was evaluated with the data from three different studies: human (Experiment 2), mannequin, and dynamics simulations. We achieved 100% sensitivity, 100% specificity, 100% accuracy, and a lead time of 301.8 ± 87.8 ms for the human study of Experiment 2.

In order to validate the results from both mannequin experiments and dynamics simulations, the VA and VV in 0.7 m jump and 0.7 m forward FFH were compared with those from human experiments (Table 5). An independent-sample *t*-test (*p* < 0.05) was conducted using IBM SPSS Statistics 25 to compare the data. In 0.7 m jump, no significant difference in the VA (roll) and VV was observed. However, a difference was found in the VA (pitch) because the upper body tended to bend over to maintain balance in human experiments. Furthermore, in 0.7 m forward FFH, a significant difference in the VA (roll), but not in the VA (pitch) and VV, was found. A slight rotation of the mannequin during forward FFH motion was found probably due to the uneven push. 

Statistical analysis could not be performed for the dynamics simulations. Some differences were observed for the VA in 0.7 m jump. Free fall from dynamics simulations differs from the actual human jump since humans tend to bend their upper body when jumping. 

When evaluating the developed algorithm using the data from the mannequin experiments, we achieved 100% accuracy and a lead time of over 130 ms. Table 6 presents the lead times obtained according to the motions performed in the mannequin experiments.

When evaluating the algorithm using the data from the dynamics simulations, we achieved 100% accuracy and a lead time of over 240 ms. Table 7 presents the lead times obtained according to different motions in the dynamics simulations.

## 4. Discussion

Dogan et al. [23] developed an algorithm for detecting FFHs with 100% accuracy using an IMU sensor. However, the study had no actual human motion data to evaluate the algorithm objectively, and it is difficult to apply the algorithm to wearable airbags because it was a post-impact FFH detection algorithm. Yang et al. [24] and Dzeng et al. [25] developed FFH detection algorithms based on the inertial data obtained from experiments with human participants, and they achieved relatively high FFH detection accuracies. However, these algorithms cannot be applied to wearable airbags because the number of participants and motions was too small, and the algorithms were not pre-impact FFH detection algorithms. 

In this study, a sufficient amount of data was obtained from 40 healthy young males in the experiments, including 16 different types of motions. In addition, data on FFHs from great heights were obtained through mannequin experiments and dynamics simulations. An algorithm for detecting pre-impact FFHs was developed with 100% accuracy based on the data obtained. The algorithm achieved sufficient lead time for wearable airbags to be inflated before collision with the floor. Table 8 shows a comparison of results from this study and those from existing studies.

### 4.1. Lead Time

When evaluating the developed algorithm using the data from Experiment 2, a lead time of 301.8 ± 87.8 ms was obtained. This lead time was smaller than that obtained using the data from Experiment 1, and the standard deviation was larger. We observed that the VA changed slightly when the knee touched the ground first in FFHs, yielding a short lead time. These results were obtained because more participants touched their knees to the ground first during FFHs in Experiment 2 than in Experiment 1.

In the mannequin experiments, the lead time of forward FFHs was longer than that of vertical FFHs at the same height. In vertical FFHs, the VA did not change. Therefore, the VV had to exceed 5 m/s to be detected as FFHs. Further, forward FFHs were detected when the VV exceeded 1.8 m/s because the VA exceeded 40°. Detecting FFHs at low VVs caused FFHs to be detected faster, resulting in longer lead times.

In the dynamics simulations, the lead time obtained for the ladder FFHs was longer than those for the scaffold and roof FFHs. Ladder FFHs were detected as FFHs at a VV of 1.8 m/s as the VA exceeded 40°, similar to forward FFHs in the mannequin experiments. However, other FFHs were detected as FFHs at a VV of 5 m/s because the VA did not exceed 40°. Thus, the ladder FFHs had longer lead times.

The lead times obtained in the experiments involving humans, in the mannequin experiments, and in the dynamics simulations were 301.8 ± 87.8 ms, greater than 130 ms, and greater than 240 ms, respectively. The inflation time of the developed wearable airbag with a non-gunpowder inflator was approximately 100 ms [15]. If the present algorithm is applied to wearable airbags, the lead time is sufficient to inflate the airbags before collision with the floor.

### 4.2. Limitations

Since FFHs from great heights could not be performed for safety issues in human experiments, mannequin experiments and dynamics simulations were performed. However, it was difficult to implement motion patterns that a human reacts in the actual FFH in mannequin experiments and dynamics simulations. Therefore, there was a difference between the actual FFH data and the experimental data. In addition, due to limited space and lack of equipment, non-FFH and FFH motions that could be performed were limited.

## 5. Conclusions

In this study, a threshold-based algorithm was developed for detecting pre-impact FFHs using only one IMU sensor. Forty healthy young males participated in two types of experiments. Data from 20 of the participants were used to develop the FFH detection algorithm, whereas data from the remaining 20 participants were used to evaluate the algorithm. In addition, mannequin experiments and dynamics simulations were performed to obtain inertial data on FFHs from great heights. The VA and VV were calculated using the inertial data from the sensor attached to the T7 and were used as parameters of the algorithm.

The algorithm achieved 100% accuracy in all the experiments. The lead times obtained during the experiments involving humans, the mannequin experiments, and the dynamics simulations were 301.8 ± 87.8 ms, greater than 130 ms, and greater than 240 ms, respectively. In the future, the algorithm will be further evaluated, improved using inertial data obtained from on-site testing, and applied to wearable airbags to significantly mitigate the damage caused by FFHs.

## Figures and Tables

**Table 1:** List of motions in Experiment 1.

Motions		
Non-FFH motions	Siting quickly and getting up	Lifting (front)
	Siting on the floor and getting up	Lifting (back)
	Climbing up and down stairs	Lifting (side)
	Climbing up and down a ladder	0.7 m jump
	Working with a pickaxe	
FFH motion	0.7 m forward FFH	

**Table 2:** List of additional motions in Experiment 2.

Motions	
Non-FFH motions	Walking on a beam
	Walking on a beam with luggage in one hand
	Shoveling
	Stretching
	Climbing up and down a scaffold
	Moving up and down in an elevator

**Table 3:** Sensitivity, specificity, and accuracy obtained using threshold combinations.

VA (°)	VV (m/s)	Sensitivity (%)	Specificity (%)	Accuracy (%)
25	2.5	100	97.04	97.33
30	2.2	100	99.63	99.67
35	1.9	100	100	100
40	1.8	100	100	100

**Table 4:** Lead times according to threshold combinations.

VA Threshold (°)	VV Threshold (m/s)	Lead Time (ms)
35	1.9	326.7 ± 41.7
40	1.8	333.7 ± 45.8

**Table 5:** VA (°) and VV (m/s) determined from experiments involving humans and the mannequin and from dynamics simulations.

		0.7 m Jump	0.7 m Forward FFHs
VA (roll)	Human	7.1 ± 2.7	11.6 ± 5.4
	Mannequin	6.0 ± 1.0	25.8 ± 7.6
	Dynamics simulation	0.3	13.7
VA (pitch)	Human	33.5 ± 13.3	87.8 ± 8.5
	Mannequin	4.6 ± 2.9	81.5 ± 3.1
	Dynamics simulation	5.9	94.6
VV	Human	3.4 ± 0.6	4.7 ± 0.4
	Mannequin	3.3 ± 0.1	4.5 ± 0.2
	Dynamics simulation	3.5	4.7

**Table 6:** Lead times (ms) during various motions in the mannequin experiments.

Height (m)	Vertical FFHs	Forward FFHs
2	176.0 ± 37.7	306.0 ± 34.4
2.5	270.0 ± 64.8	378.0 ± 44.0
3	368.0 ± 20.4	472.0 ± 31.9

**Table 7:** Lead times (ms) according to different motions in the dynamics simulations.

	3 m	4.5 m	6 m
Scaffold FFHs	280	440	560
Roof FFHs	240	410	560
Ladder FFHs	460	640	780

**Table 8:** Comparison with other studies.

	Dogan et al. [23] (2019)	Yang et al. [24] (2014)	Dzeng et al. [25] (2017)	This Study
**Participants**	X	2 males	3 males, 1 female	40 males
**Sensor**	IMU	IMU (waist)	IMU (back waist)	IMU (T7)
**Method**	Threshold (Acc RMS)	Machine learning (SVM)	Threshold (Acc RMS)	Threshold (VA, VV)
**Motions**	1 type (free fall)	6 types (near-miss fall)	6 types (1 FFH)	16 types (1 FFH)
**Accuracy**	100%	93.8%	96.6%	100%
**Lead time**	X	X	X	301 ms
